# Supplementary material for: Forecasting of influenza activity and associated hospital admission burden and estimating the impact of COVID-19 pandemic on 2019/20 winter season in Hong Kong
Source: PLoS Comput Biol. 2024 Jul 31;20(7):e1012311. doi: 10.1371/journal.pcbi.1012311 (PMC11318919; doi:10.1371/journal.pcbi.1012311)
Supplement: S1 Text — (PDF) [file pcbi.1012311.s001.pdf]

Supplementary for Forecasting of influenza activity and associated hospital admission burden and estimating the impact of COVID-19 pandemic on 2019/2020 winter season in Hong Kong

AUTHORS:

Yiu-Chung Lau[1,2], Songwei Shan[1,2], Dong Wang[1,2], Dongxuan Chen[1,2], Zhanwei Du[1,2], Eric H. Y. Lau[1,2,3], Daihai He[4], Linwei Tian[1], Peng Wu[1,2], Benjamin J. Cowling[1,2,*], Sheikh Taslim Ali[1,2]

AFFILIATIONS:

[1] WHO Collaborating Centre for Infectious Disease Epidemiology and Control, School of Public Health, Li Ka Shing Faculty of Medicine, The University of Hong Kong, Hong Kong Special Administrative Region, China

[2] Laboratory of Data Discovery for Health Limited, Hong Kong Science Park, New Territories, Hong Kong Special Administrative Region, China

[3] Institute for Health Transformation, School of Health and Social Development, Deakin University, Burwood, Australia

[4] Department of Applied Mathematics, Hong Kong Polytechnic University, Hong Kong Special Administrative Region, China

* bcowling@hku.hk

Supplementary Tables

Table A. Short-term (1-4 weeks) forecasted maximum influenza incidence from cross-validated models under the counterfactual scenario without COVID-19 pandemic waves in the winter period of season 2019/2020. The forecasts were based on the models with the lowest cross-validated cost values. Forecasts were updated by week since the first week of January 2020 until the first COVID case was found before the last week of January. PI: prediction interval; WIS: weighted interval score; RMSE: root mean squared error; RMSLE: root mean squared log error; MAE: mean absolute error

**Estimates, % (95% PI)**	**Forecast Since**			
**Model**	**1st week of Jan 2020**	**2nd week of Jan 2020**	**3rd week of Jan 2020**	**4th week of Jan 2020**
WIS	1.0 (0.2 - 2.3)	1.1 (0.2 - 2.6)	1.3 (0.3 - 3.0)	1.0 (0.2 - 2.3)
RMSE / MAE	0.8 (0.2 - 2.0)	1.0 (0.2 - 2.3)	1.0 (0.2 - 2.3)	0.8 (0.3 - 1.7)
RMSLE	0.8 (0.2 - 1.9)	1.0 (0.2 - 2.4)	1.1 (0.2 - 2.6)	1.0 (0.2 - 2.2)

Table B. Medium-term (1-13 weeks) forecasted influenza incidence, attack rate and reduction in attack rate from cross-validated models under the counterfactual scenario without COVID-19 pandemic waves in the winter period of season 2019/2020. The forecasts were based on the models with the lowest cross-validated cost values. Forecasts were updated by week since the first week of January 2020 until the first COVID case was found before the last week of January. Attack rate was defined as the cumulative sum of weekly incidence in the winter-spring period (December 2019 – March 2020), where the reduction in attack rate was defined as the percentage reduction between the observed and the forecasted attack rate. ^PI: prediction interval; *CI: confidence interval; WIS: weighted interval score; RMSE: root mean squared error; RMSLE: root mean squared log error; MAE: mean absolute error

**Estimates, % (95% ^PI / *CI)**		**Forecast Since**			
**Model**		**1st week of Jan 2020**	**2nd week of Jan 2020**	**3rd week of Jan 2020**	**4th week of Jan 2020**
WIS	Peak Time	23rd – 29th February	26th January – 1st February	2nd February – 8th February	9th – 15th February
	Peak Incidence^	1.2 (0.1 - 3.6)	1.1 (0.2 - 2.6)	1.2 (0.2 - 2.8)	0.9 (0.2 - 2.3)
	Reduction in mean peak incidence*	40.9 (-1.5 - 58.3)	36.4 (6.7 - 51.8)	41.5 (13.9 - 55.7)	26.9 (-3.4 - 43.5)
	Attack rate^	12.0 (7.8 - 17.4)	10.9 (7.3 - 15.2)	11.9 (8.3 - 16.5)	10.3 (7.4 - 13.8)
	Reduction in attack rate*	75.7 (72.7 - 78.1)	73.2 (70.3 - 75.7)	75.5 (73.0 - 77.6)	71.9 (69.5 - 73.9)
RMSE	Peak Time	23rd – 29th February	26th January – 1st February	2nd February – 8th February	26th January – 1st February
	Peak Incidence^	1.1 (0.1 - 3.4)	1.1 (0.2 - 2.7)	1.2 (0.2 - 2.8)	0.9 (0.3 - 1.9)
	Reduction in mean peak incidence*	38.6 (-6.9 - 57.0)	38.3 (9.1 - 53.3)	42.1 (14.8 - 56.2)	26.9 (6.6 - 39.9)
	Attack rate^	11.6 (7.5 - 16.7)	11.1 (7.5 - 15.5)	11.9 (8.4 - 16.3)	10.3 (7.4 - 13.8)
	Reduction in attack rate*	74.9 (71.7 - 77.5)	73.8 (70.8 - 76.2)	75.6 (73.1 - 77.7)	71.6 (69.2 - 73.7)
RMSLE	Peak Time	23rd – 29th February	26th January – 1st February	2nd February – 8th February	16th -22nd February
	Peak Incidence^	1.2 (0.1 - 3.4)	1.3 (0.3 - 3.2)	1.1 (0.2 - 2.6)	0.9 (0.2 - 2.3)
	Reduction in mean peak incidence*	40.5 (2.7 - 57.2)	47.9 (23.3 - 60.6)	35.3 (9.5 - 49.6)	22.3 (-6.9 - 39.0)
	Attack rate^	11.7 (7.6 - 16.9)	12.3 (8.3 - 17.4)	10.8 (7.5 - 14.8)	9.7 (7.0 - 13.0)
	Reduction in attack rate*	75.1 (72.2 - 77.4)	76.4 (73.7 - 78.6)	73.0 (70.6 - 75.1)	69.9 (67.7 - 71.7)
MAE	Peak Time	23rd – 29th February	26th January – 1st February	2nd February – 8th February	9th – 15th February
	Peak Incidence^	1.2 (0.1 - 3.5)	1.2 (0.3 - 3.0)	1.2 (0.3 - 2.9)	0.9 (0.2 - 2.2)
	Reduction in mean peak incidence*	40.7 (-1.6 - 58.1)	44.0 (16.9 - 57.8)	43.9 (17.7 - 57.5)	26.3 (-4.5 - 43.1)
	Attack rate^	12.0 (7.9 - 17.2)	11.7 (7.9 - 16.5)	12.2 (8.5 - 16.8)	10.3 (7.5 - 13.8)
	Reduction in attack rate*	75.8 (72.9 - 78.2)	75.2 (72.4 - 77.5)	76.2 (73.7 - 78.2)	71.8 (69.4 - 73.8)

Table C. Effective reproduction number under the counterfactual scenario without COVID-19 pandemic waves in the winter period of season 2019/2020. The instantaneous effective reproduction number under the counterfactual scenario was calculated based on the forecast under the cross-validated WIS model. CrI: credible interval. ^Based on prediction uncertainty; *Based on prediction mean uncertainty

		Forecast Since							
**Estimates (95% CrI)**		**1st week of Jan 2020**		**2nd week of Jan 2020**		**3rd week of Jan 2020**		**4th week of Jan 2020**	
**Week**	**Observed**	**Counterfactual^**	**Reduction***	**Counterfactual^**	**Reduction***	**Counterfactual^**	**Reduction***	**Counterfactual^**	**Reduction***
**29 Dec 2019 – 4 Jan 2020**	1.37 (1.35, 1.38)	1.22 (0.85, 1.65)	-14.16% (-23.98%, -6.93%)	1.19 (0.94, 1.47)	-13.03% (-18.88%, -7.63%)	1.36 (1.33, 1.39)	-0.96% (-1.10%, -0.68%)	1.36 (1.34, 1.37)	-0.85% (-0.86%, -0.84%)
**5 Jan 2020 – 11 Jan 2020**	1.08 (1.07, 1.09)	1.19 (0.78, 1.74)	10.91% (3.47%, 19.04%)	1.19 (0.83, 1.60)	9.66% (4.33%, 16.65%)	1.15 (0.91, 1.42)	8.30% (4.99%, 10.16%)	1.17 (1.15, 1.19)	7.53% (7.29%, 7.79%)
**12 Jan 2020 – 18 Jan 2020**	0.77 (0.77, 0.78)	1.12 (0.68, 1.69)	28.96% (21.45%, 36.04%)	1.18 (0.77, 1.69)	31.81% (24.92%, 36.99%)	1.08 (0.77, 1.45)	27.82% (22.23%, 32.68%)	1.01 (0.80, 1.24)	24.02% (21.14%, 26.49%)
**19 Jan 2020 – 25 Jan 2020**	0.91 (0.89, 0.92)	1.04 (0.62, 1.65)	14.28% (6.50%, 22.38%)	1.05 (0.65, 1.57)	15.83% (9.34%, 20.12%)	1.04 (0.69, 1.48)	9.01% (-2.16%, 21.55%)	1.01 (0.72, 1.35)	10.14% (2.38%, 14.97%)
**26 Jan 2020 – 1 Feb 2020**	0.87 (0.85, 0.88)	1.04 (0.59, 1.68)	15.36% (-4.14%, 24.42%)	1.00 (0.60, 1.57)	11.57% (4.34%, 21.18%)	1.02 (0.62, 1.53)	15.58% (4.35%, 22.13%)	1.01 (0.67, 1.45)	14.77% (7.19%, 21.87%)
**2 Feb 2020 – 8 Feb 2020**	0.69 (0.67, 0.70)	1.04 (0.57, 1.73)	30.59% (23.72%, 35.87%)	0.99 (0.57, 1.62)	29.54% (20.90%, 35.01%)	0.99 (0.59, 1.55)	31.98% (24.51%, 37.58%)	1.01 (0.62, 1.52)	31.82% (23.51%, 38.19%)
**9 Feb 2020 – 15 Feb 2020**	0.53 (0.50, 0.55)	1.02 (0.55, 1.75)	48.34% (41.01%, 55.97%)	0.96 (0.54, 1.62)	44.50% (38.30%, 50.79%)	0.98 (0.56, 1.61)	44.59% (38.57%, 51.88%)	1.00 (0.59, 1.58)	46.73% (39.47%, 53.29%)
**16 Feb 2020 – 22 Feb 2020**	0.77 (0.72, 0.83)	1.03 (0.54, 1.83)	26.40% (20.90%, 36.44%)	1.02 (0.53, 1.74)	23.76% (14.20%, 29.51%)	1.02 (0.54, 1.71)	24.99% (12.58%, 33.46%)	0.99 (0.57, 1.61)	19.19% (8.14%, 30.14%)
**23 Feb 2020 – 29 Feb 2020**	0.75 (0.68, 0.82)	1.01 (0.51, 1.80)	21.31% (0.63%, 35.22%)	1.02 (0.53, 1.78)	26.27% (12.38%, 37.47%)	1.01 (0.53, 1.72)	25.47% (10.88%, 40.06%)	1.01 (0.54, 1.71)	25.12% (11.40%, 35.78%)

Table D. Prediction performance of models on influenza-associated hospital admission rate based on time series cross-validation. The models with the lowest cross-validated weighted interval score (WIS), root mean square error (RMSE), root mean square log error (RMSLE), and mean absolute error (MAE) were shown. The model with the lowest WIS was selected for further forecasting (bolded). The superscript 2 indicates the inclusion of both quadratic and linear terms of the corresponding covariate in the model. H(n): lagged influenza-associated hospital admission rate up to n weeks; AH: absolute humidity; Temp: temperature; Ozone: Ozone concentration; School: School holiday/closure

**Rank (cost value)**	**Model**	**WIS**	**RMSE**	**RMSLE**	**MAE**
Short-term (1-4 weeks)	**H(10) + monthly spline + log(AH) + log(Temp) + School**	**1 (6.1)**	30 (10.7)	179 (0.4)	20 (9.4)
	H(10) + monthly spline + log(AH) + $Temp^2$ + log(School)	20 (6.1)	1 (10.6)	360 (0.4)	3 (9.3)
	H(10) + monthly spline + log(AH) + log(Ozone) + log(Temp) + log(School)	673 (6.4)	354 (11.1)	1 (0.4)	412 (9.9)
	H(10) + monthly spline + AH + Temp + log(School)	12 (6.1)	4 (10.6)	107 (0.4)	1 (9.2)
Medium-term (1-13 weeks)	**H(9) + monthly spline + AH + $Temp^2$ + School**	**1 (8.0)**	4 (16.4)	49 (0.7)	1 (12.9)
	H(9) + monthly spline + $Temp^2$ + log(School)	3 (8.0)	1 (16.4)	84 (0.7)	2 (12.9)
	H(7) + monthly spline + log(AH) + $Temp^2$	106 (8.1)	159 (16.8)	1 (0.7)	179 (13.2)

Table E. Short-term (1-4 weeks) forecasted maximum influenza-associated hospital admission rates from cross-validated models under the counterfactual scenario without COVID-19 pandemic waves in the winter period of season 2019/2020. The forecasts were based on the models with the lowest cross-validated cost values. Forecasts were updated by week since the first week of January 2020 until the first COVID case was found before the last week of January. PI: prediction interval; WIS: weighted interval score; RMSE: root mean squared error; RMSLE: root mean squared log error; MAE: mean absolute error

**Estimates, % (95% PI)**	**Forecast Since**			
**Model**	**1st week of Jan 2020**	**2nd week of Jan 2020**	**3rd week of Jan 2020**	**4th week of Jan 2020**
WIS	0.8 (0.2 - 1.8)	1.4 (0.4 - 3.2)	1.3 (0.5 - 2.5)	1.0 (0.3 - 2.2)
RMSE	0.8 (0.2 - 1.8)	1.4 (0.4 - 3.1)	1.3 (0.5 - 2.6)	1.1 (0.3 - 2.3)
RMSLE	0.8 (0.2 - 1.7)	1.4 (0.4 - 3.0)	1.2 (0.5 - 2.4)	1.0 (0.3 - 2.1)
MAE	0.8 (0.2 - 1.7)	1.4 (0.4 - 3.1)	1.3 (0.5 - 2.5)	1.0 (0.3 - 2.2)

Table F. Medium-term (1-13 weeks) forecasted influenza-associated hospital admission rates, cumulative admission rates and reduction in cumulative admission rate from cross-validated models under the counterfactual scenario without COVID-19 pandemic waves in the winter period of season 2019/2020. The forecasts were based on the models with the lowest cross-validated cost values. Forecasts were updated by week since the first week of January 2020 until the first COVID case was found before the last week of January. ^PI: prediction interval; *CI: confidence interval; WIS: weighted interval score; RMSE: root mean squared error; RMSLE: root mean squared log error; MAE: mean absolute error

**Estimates, % (95% ^PI / *CI)**		**Forecast Since**			
**Model**		**1st week of Jan 2020**	**2nd week of Jan 2020**	**3rd week of Jan 2020**	**4th week of Jan 2020**
**WIS / MAE**	Peak Time	26th January -1st February	26th January -1st February	9th – 15th February	16th – 22nd February
	Peak admission rate^	0.9 (0.2 – 2.1)	1.5 (0.4 – 3.3)	1.4 (0.3 – 3.2)	1.1 (0.2 – 2.6)
	Reduction in mean peak admission rate*	-3.8 (-60.1 – 23.2)	39.8 (9.8 – 54.8)	32.2 (-5.6 – 50.1)	15.3 (-20.9 – 34.9)
	Cumulative admission rate^	9.2 (6.5 – 12.4)	12.4 (9.1 – 16.5)	12.9 (9.6 – 16.8)	11.1 (8.7 – 13.9)
	Reduction in cumulative admission rate*	48.1 (42.5 – 52.7)	61.8 (57.6 – 65.3)	63.1 (59.3 – 66.3)	57.1 (53.9 – 59.9)
**RMSE**	Peak Time	26th January -1st February	26th January -1st February	9th – 15th February	9th – 15th February
	Peak admission rate^	0.9 (0.2 – 2.1)	1.3 (0.4 – 3.0)	1.3 (0.3 – 3.1)	1.2 (0.3 – 2.5)
	Reduction in mean peak admission rate*	-6.3 (-65.7 – 21.8)	31.0 (-1.0 – 47.6)	31.0 (-8.0 – 49.3)	19.4 (-11.8 – 37.0)
	Cumulative admission rate^	9.0 (6.4 – 12.2)	11.7 (8.6 – 15.6)	12.8 (9.6 – 16.7)	11.4 (8.9 – 14.4)
	Reduction in cumulative admission rate*	47.5 (41.7 – 52.2)	59.5 (55.4 – 63.0)	62.8 (59.0 – 66.0)	58.2 (55.2 – 60.8)
**RMSLE**	Peak Time	26th January -1st February	26th January -1st February	9th – 15th February	16th – 22nd February
	Peak admission rate^	0.9 (0.2 – 2.1)	1.5 (0.4 – 3.3)	1.4 (0.3 – 3.2)	1.1 (0.2 – 2.6)
	Reduction in mean peak admission rate*	-3.8 (-60.1 – 23.2)	39.8 (9.8 – 54.8)	32.2 (-5.6 – 50.1)	15.3 (-20.9 – 34.9)
	Cumulative admission rate^	9.2 (6.5 – 12.4)	12.4 (9.1 – 16.5)	12.9 (9.6 – 16.8)	11.1 (8.7 – 13.9)
	Reduction in cumulative admission rate*	48.1 (42.5 – 52.7)	61.8 (57.6 – 65.3)	63.1 (59.3 – 66.3)	57.1 (53.9 – 59.9)

Table G. Comparison in prediction performance of models on influenza activity between GLM and ARIMA models. In additional to Table 1, the prediction performance of ARIMA models were shown, while those selected ARIMA models were the 5 most popular models adopted throughout the training period. The performance was represented in ratio relative to the minimum cost value obtained across the models by cost function. The table was ordered by WIS, and the WIS-based GLM reported in main text was in bold. The superscript 2 indicates the inclusion of both quadratic and linear terms of the corresponding covariate in the model. ILI(n): lagged ILI+ proxy up to n weeks; AH: absolute humidity; Temp: temperature; Ozone: Ozone concentration; School: School holiday/closure

Ratio	Model	WIS	RMSE	RMSLE	MAE
Short-term (1-4 weeks)	**ILI(23) + monthly spline + log(Ozone) + log(School)**	1	1.005	1.018	1.006
	ILI(23) + monthly spline + log(Ozone) + Temp + log(School)	1.001	1	1.016	1
	ILI(9) + monthly spline + log(Ozone) + Temp + log(School)	1.028	1.039	1	1.04
	ARIMA(4,0,1)(0,0,0)$_{52}$ + monthly spline + log(Ozone) + Temp + log(School)	1.094	1.052	1.004	1.069
	ARIMA(2,0,2)(1,0,0)$_{52}$ + monthly spline + log(Ozone) + Temp + log(School)	1.127	1.079	1.012	1.096
	ARIMA(2,0,2)(1,0,0)$_{52}$ + log(Ozone) + Temp + log(School)	1.130	1.104	1.100	1.119
	ARIMA(2,0,2)(0,0,0)$_{52}$ + log(Ozone) + Temp + log(School)	1.139	1.113	1.109	1.128
	ARIMA(2,0,2)(0,0,0)$_{52}$ + monthly spline + log(Ozone) + Temp + log(School)	1.142	1.095	1.031	1.115
Medium-term (1-13 weeks)	**ILI(15) + monthly spline + Temp + School^2**	1	1.001	1.092	1.013
	ILI(17) + monthly spline + Temp + School^2	1.003	1	1.098	1.015
	ILI(15) + monthly spline + AH + Temp + School^2	1.003	1.004	1.085	1.013
	ILI(5) + monthly spline + AH + Temp + School^2	1.016	1.023	1.078	1.037
	ARIMA(4,0,1)(0,0,0)$_{52}$ + monthly spline + AH + Temp + School^2	1.077	1.009	1	1
	ARIMA(2,0,2)(1,0,0)$_{52}$ + monthly spline + AH + Temp + School^2	1.112	1.034	1.016	1.034
	ARIMA(2,0,2)(0,0,0)$_{52}$ + monthly spline + AH + Temp + School^2	1.155	1.061	1.050	1.072
	ARIMA(2,0,2)(1,0,0)$_{52}$ + AH + Temp + School^2	1.209	1.137	1.180	1.121
	ARIMA(2,0,2)(0,0,0)$_{52}$ + AH + Temp + School^2	1.231	1.153	1.206	1.138

Table H. Comparison in prediction performance of models on influenza-associated hospitalization rate between GLM and ARIMA models. In additional to S4 Table, the prediction performance of ARIMA models were shown, while those selected ARIMA models were the 5 most popular models adopted throughout the training period. The performance was represented in ratio relative to the minimum cost value obtained across the models by cost function. The table was ordered by WIS, and the WIS-based GLM reported in main text was in bold. The superscript 2 indicates the inclusion of both quadratic and linear terms of the corresponding covariate in the model. ILI(n): lagged ILI+ proxy up to n weeks; AH: absolute humidity; Temp: temperature; Ozone: Ozone concentration; School: School holiday/closure

**Ratio**	**Model**	**WIS**	**RMSE**	**RMSLE**	**MAE**
Short-term (1-4 weeks)	**ILI(10) + monthly spline + log(AH) + log(Temp) + School**	1	1.011	1.011	1.012
	ARIMA(2,0,1)(1,0,0)$_{52}$ + monthly spline + log(AH) + log(Temp) + log(Ozone) + School	1.001	1.000	1.000	1.019
	ILI(10) + monthly spline + AH + Temp + log(School)	1.004	1.002	1.009	1
	ILI(10) + monthly spline + log(AH) + Temp^2 + log(School)	1.005	1	1.016	1.002
	ILI(10) + monthly spline + log(AH) + log(Temp) + log(Ozone) + log(School)	1.054	1.05	1	1.065
	ARIMA(2,1,2)(1,0,0)$_{52}$ + log(AH) + log(Temp) + log(Ozone) + School	1.388	1.273	1.141	1.294
	ARIMA(2,1,0)(1,0,0)$_{52}$ + log(AH) + log(Temp) + log(Ozone) + School	1.400	1.290	1.121	1.327
	ARIMA(2,1,1)(1,0,0)$_{52}$ + log(AH) + log(Temp) + log(Ozone) + School	1.453	1.345	1.112	1.375
	ARIMA(1,1,1)(1,0,0)$_{52}$ + log(AH) + log(Temp) + log(Ozone) + School	1.489	1.381	1.107	1.409
Medium-term (1-13 weeks)	ARIMA(2,0,1)(1,0,0)$_{52}$ + monthly spline + AH + Temp^2 + School	1	1	1.040	1
	**ILI(9) + monthly spline + AH + Temp^2 + School**	1.010	1.057	1.053	1.047
	ILI(9) + monthly spline + Temp^2 + log(School)	1.011	1.057	1.055	1.050
	ILI(7) + monthly spline + log(AH) + Temp^2	1.023	1.079	1.046	1.074
	ARIMA(2,0,1)(1,0,0)$_{52}$ + AH + Temp^2 + School	1.154	1.131	1	1.135
	ARIMA(2,1,0)(1,0,0)$_{52}$ + AH + Temp^2 + School	2.557	1.753	1.348	1.914
	ARIMA(2,1,1)(1,0,0)$_{52}$ + AH + Temp^2 + School	3.048	1.951	1.370	2.142
	ARIMA(1,1,1)(1,0,0)$_{52}$ + AH + Temp^2 + School	3.085	2.021	1.389	2.217

Supplementary Figures

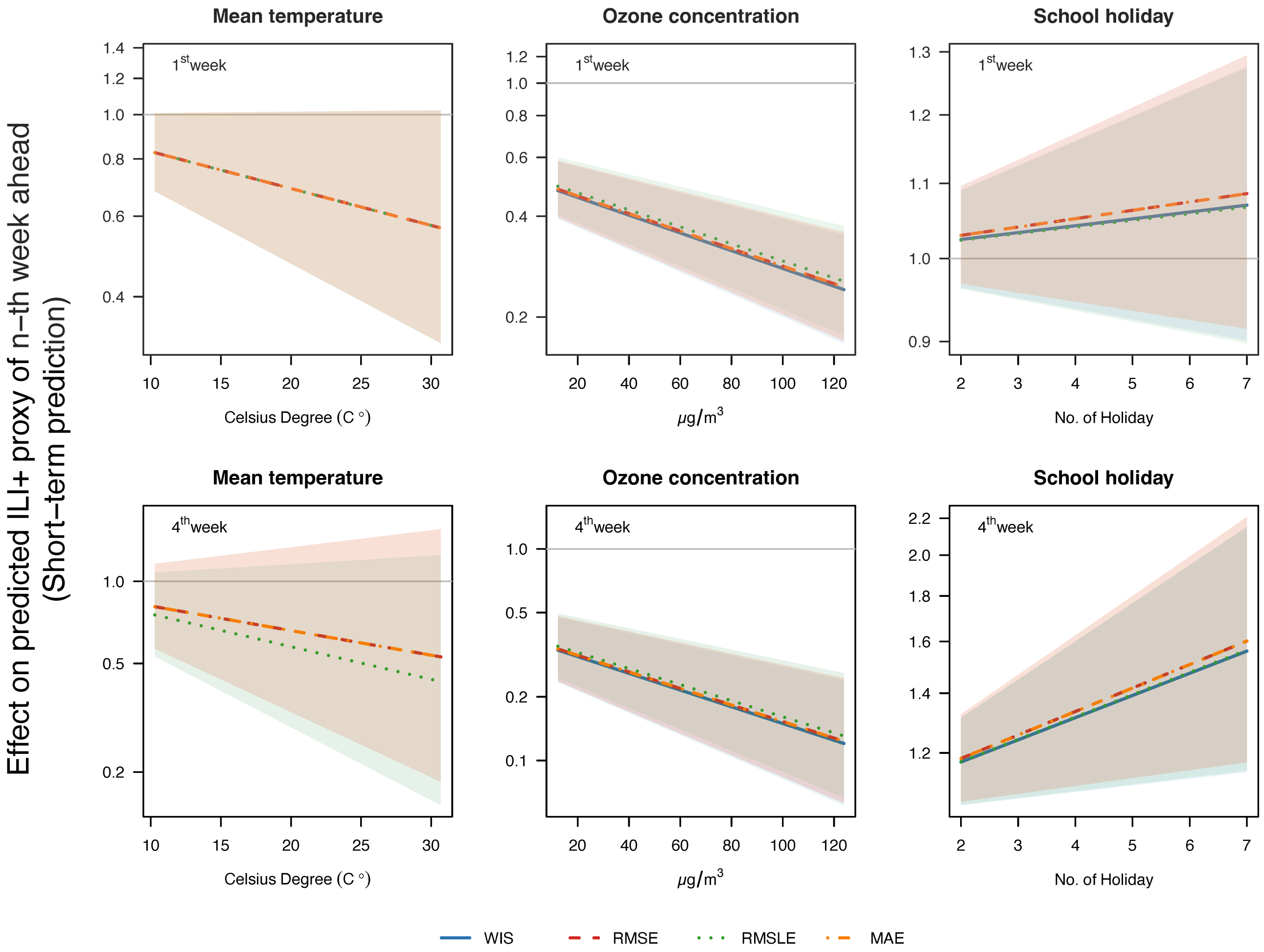

Fig A. Predictive models based evaluation of the association between drivers and influenza activity for short-term forecasting. Effect of the significant drivers on predicted ILI+ proxy 1st and 4th week ahead in short-term forecasting for respective measure of predictive model construction. The type of lines and respective colours are for the models under different measures (WIS, RMSE, RMSLE and MAE).

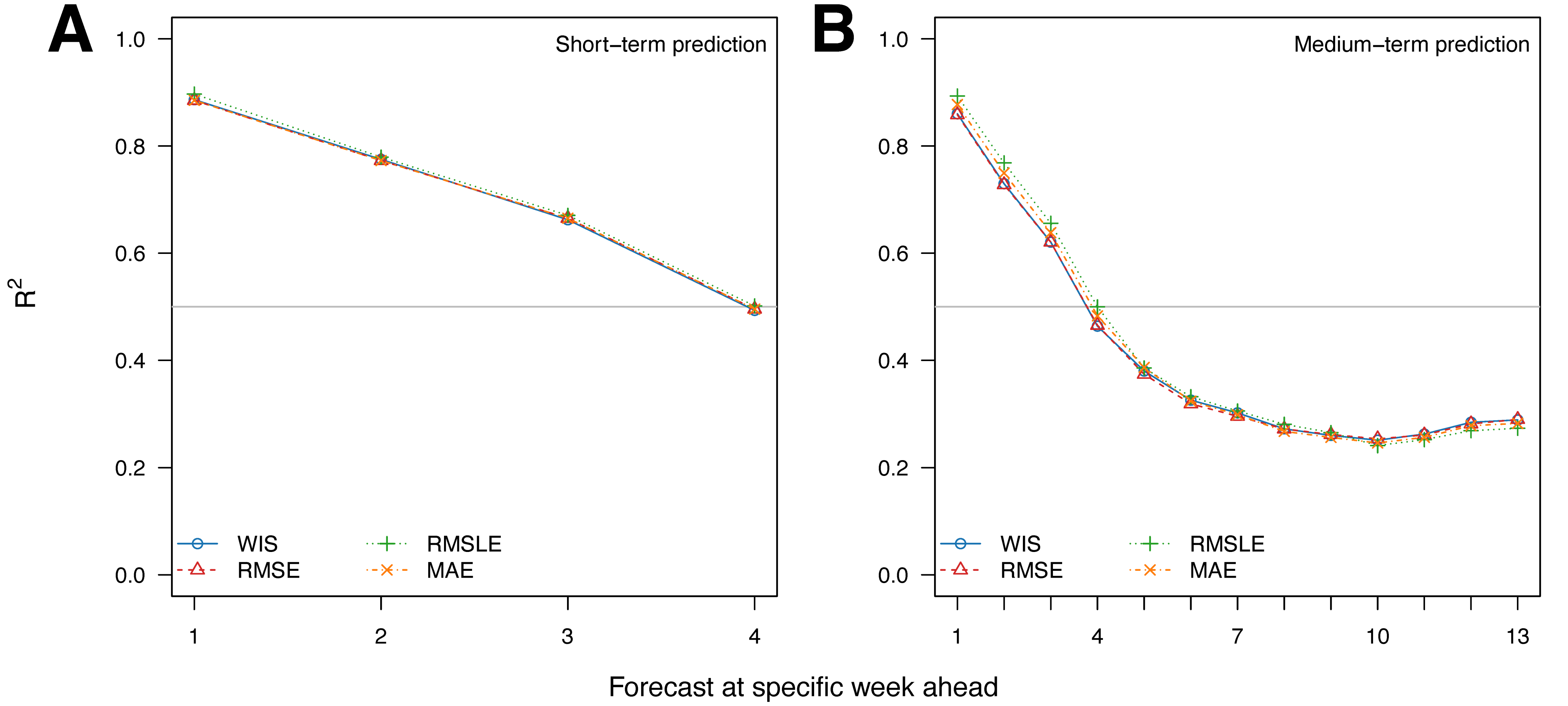

Fig B. Coefficient of determinant (R^2) between predicted and observed ILI+ proxy in each week ahead for (A) short-term (1-4 weeks ahead) and (B) medium-term (1-13 weeks ahead) prediction based on the models under different measures (WIS, RMSE, RMSLE, MAE).

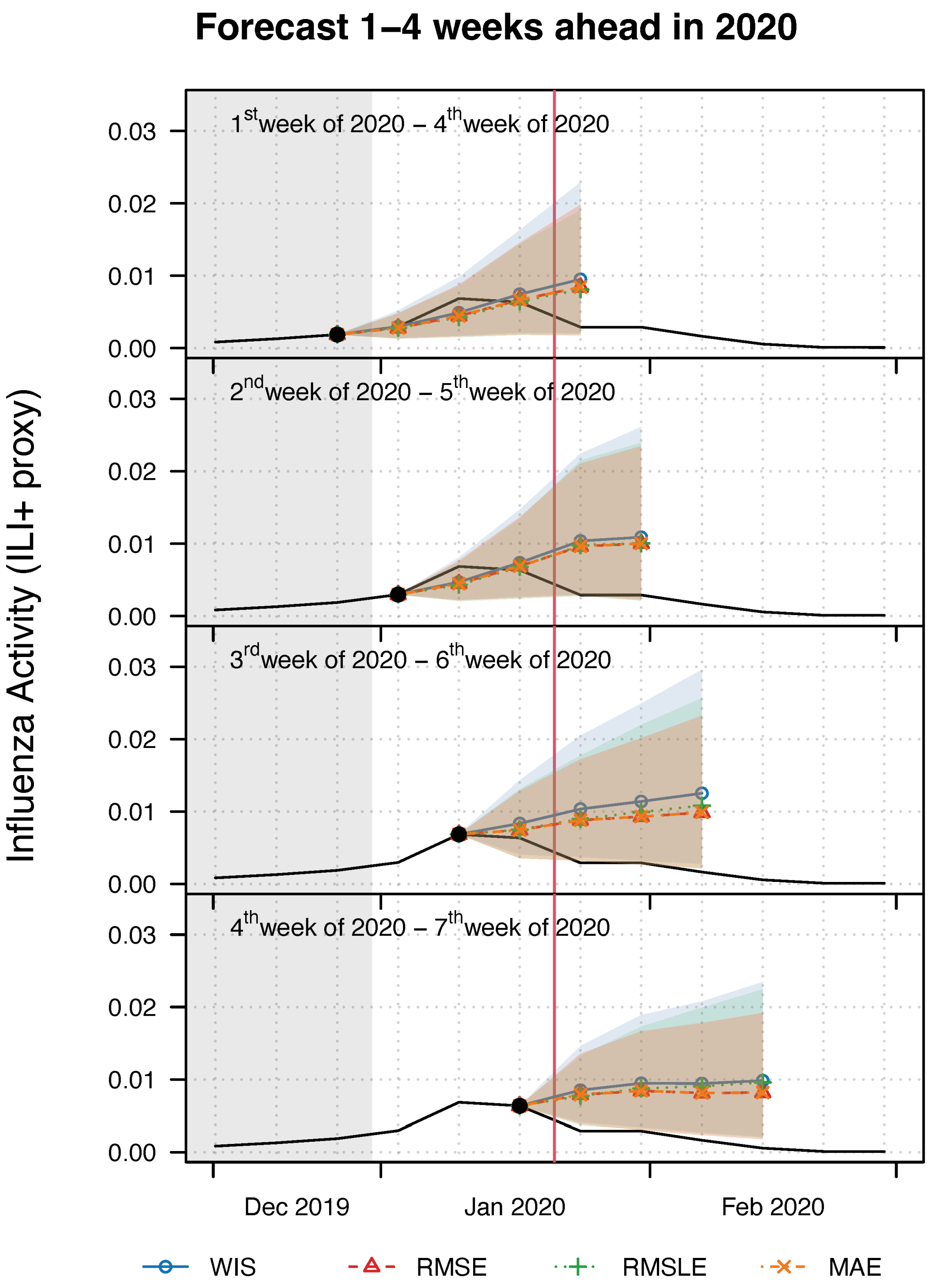

Fig C. Short-term (1-4 weeks ahead) forecast on influenza activity with different start points of forecast weeks in Hong Kong. We considered by 2nd week of January 2020, there were changes in the population behaviour at individual and community levels and the implication of PHSMs became much effective (1) , therefore we started forecast from the following weeks (for main text) and for sensitivity we consider forecasting from 1st to 4th week of January 2020. The black line is the observed ILI+ proxy and the coloured dashed lines (with respective shapes for different measures of predictive model evaluation) are the influenza activity forecast with 95% PI (in respective shades). The black dots indicated the previous week of the start points of the forecast week and the periods specified at the top-left corner in each panel were the forecast period. The vertical line in red indicates that the first COVID-19 case was identified in Hong Kong on 22nd January 2020.

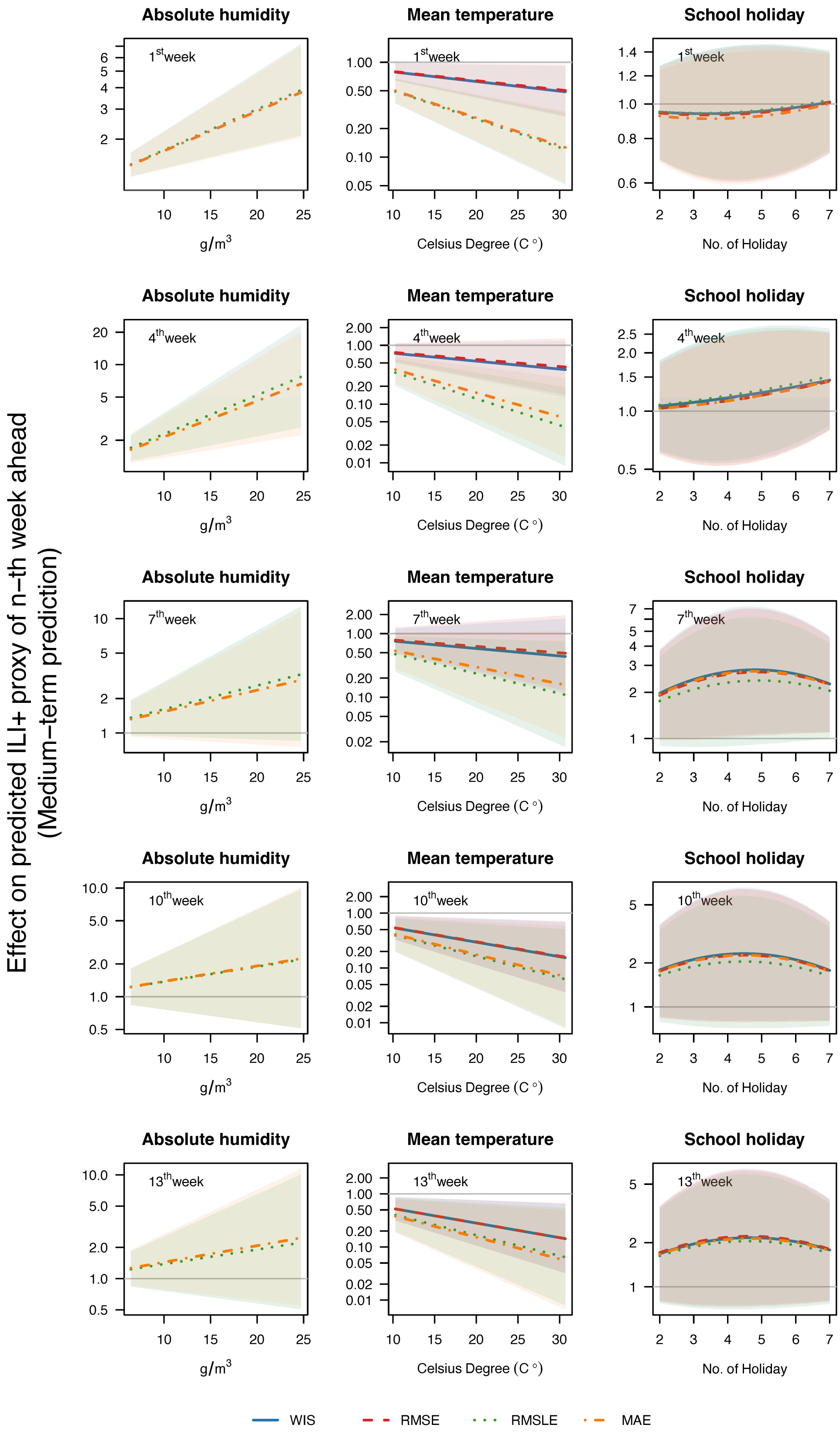

Fig D. Predictive models-based evaluation of the association between drivers and influenza activity for medium-term forecasting. Effects of the significant drivers on predicted ILI+ proxy 1,4,7,10 and 13 weeks ahead in medium-term forecasting for respective measure of predictive model construction. The type of lines and respective colours are for the models under different measures (WIS, RMSE, RMSLE and MAE).

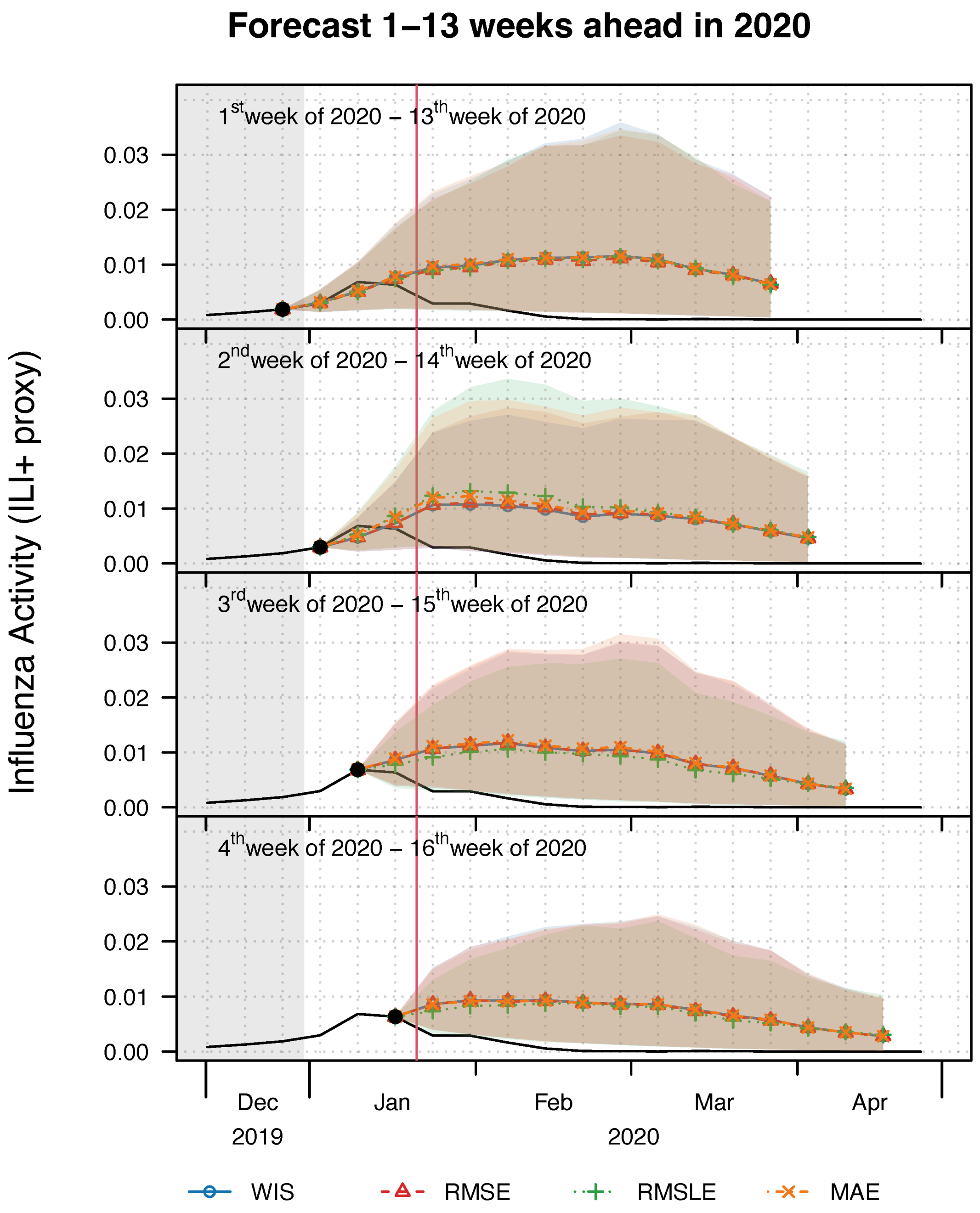

Fig E. Medium-term (1-13 weeks ahead) forecast on influenza activity with different start points of forecast weeks in Hong Kong. We considered by 2nd week of January 2020, there were changes in the population behaviour at individual and community levels and the implication of PHSMs became much effective (1) , therefore we started forecast from the following weeks (for main text) and for sensitivity we consider forecasting from 1st to 4th week of January 2020. The black line is the observed ILI+ proxy and coloured dashed lines (with respective shapes for different measures of predictive model evaluation) are the influenza activity forecast with 95% PI (in respective shades). The black dots indicated the previous week of the start points of forecast weeks and the periods specified at the top-left corner of each panel were the forecast period. The vertical line in red indicates that the first COVID-19 case was identified in Hong Kong on 22nd January 2020.

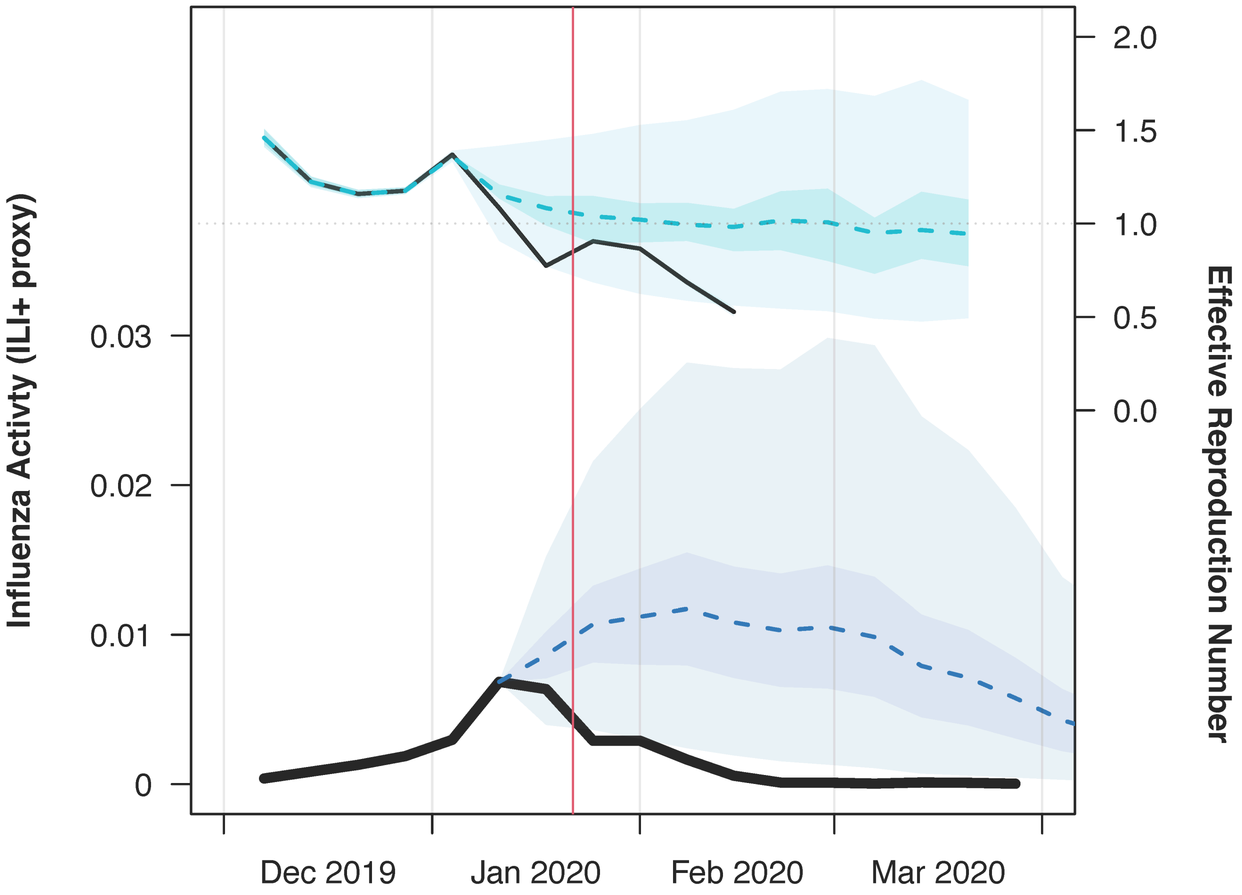

Fig F. Effective reproduction number under the counterfactual scenario without COVID-19 pandemic waves in the winter period of season 2019/2020. The instantaneous effective reproduction number under the counterfactual scenario was calculated based on the WIS model and the forecast since the 2nd week of January 2020 which yielded the highest mean peak incidence over the forecasts obtained in Table S2. The black bolded line was the observed ILI+ proxy and the blue dashed line was the influenza activity forecast with 95% confidence interval (pale blue region) and 95% prediction interval (lighter blue region). The black line showed the effective reproduction number of the observed ILI+ proxy, while the cyan dashed line showed the effective reproduction number under the counterfactual scenario with a 95% credible interval derived from a 95% confidence interval (pale cyan region), and with 95% credible interval derived from 95% prediction interval (lighter cyan region).

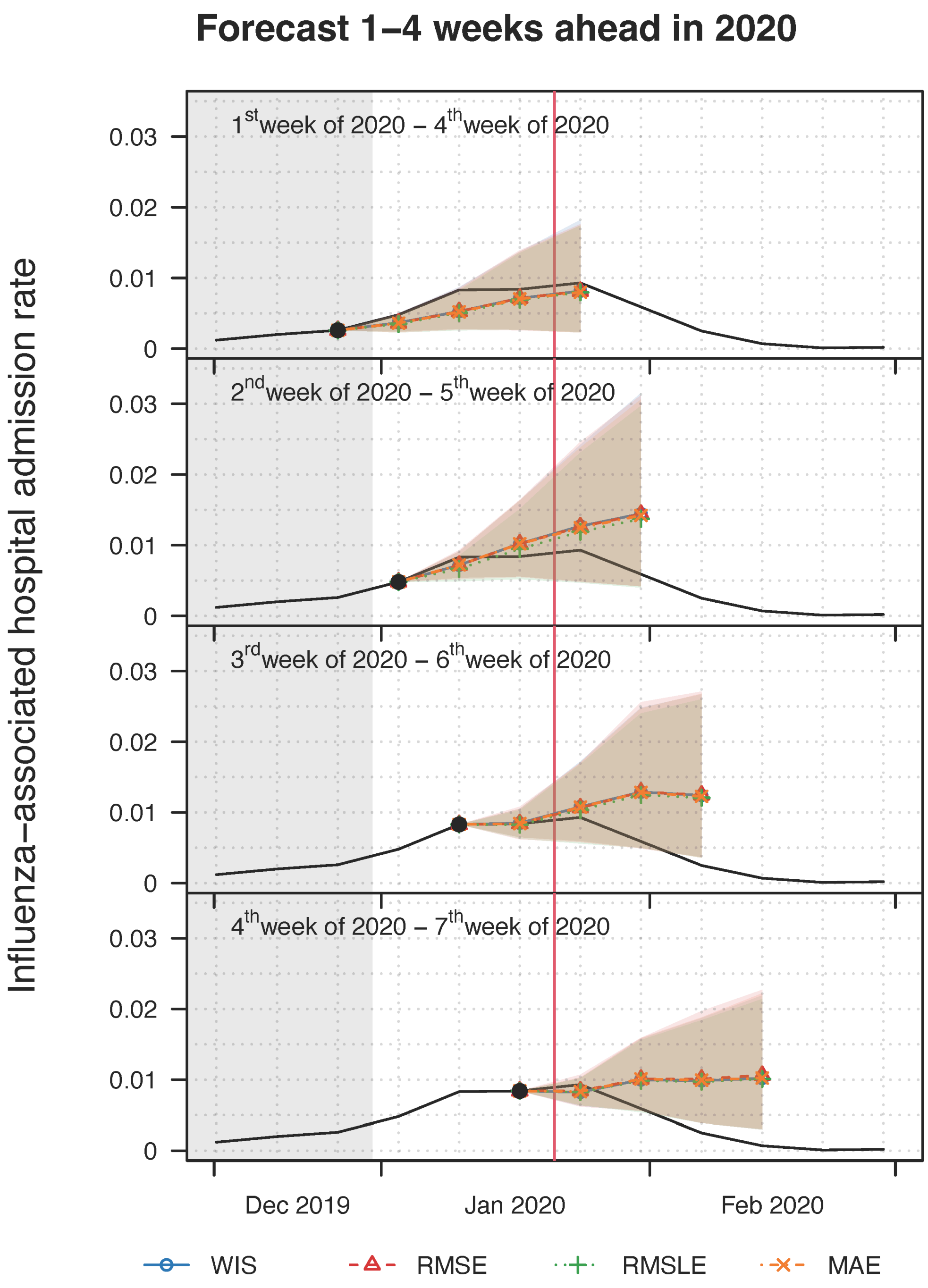

Fig G. Short-term (1-4 weeks ahead) forecast on influenza-associated hospital admission rates with different start points of forecast weeks in Hong Kong. We considered by 2nd week of January 2020, there were changes in the population behaviour at individual and community levels and the implication of PHSMs became much effective (1) , therefore we started forecast from the following weeks (for main text) and for sensitivity we consider forecasting from 1st to 4th week of January 2020. The black line is the observed influenza-associated hospital admission rate and the coloured dashed lines (with respective shapes for different measures of predictive model evaluation) are the admission rate forecast with 95% PI (in respective shades). The black dots indicated the previous week of the start points of forecast weeks and the periods specified at the top-left corner of each panel were the forecast period. The vertical line in red indicates that the first COVID-19 case was identified in Hong Kong on 22nd January 2020.

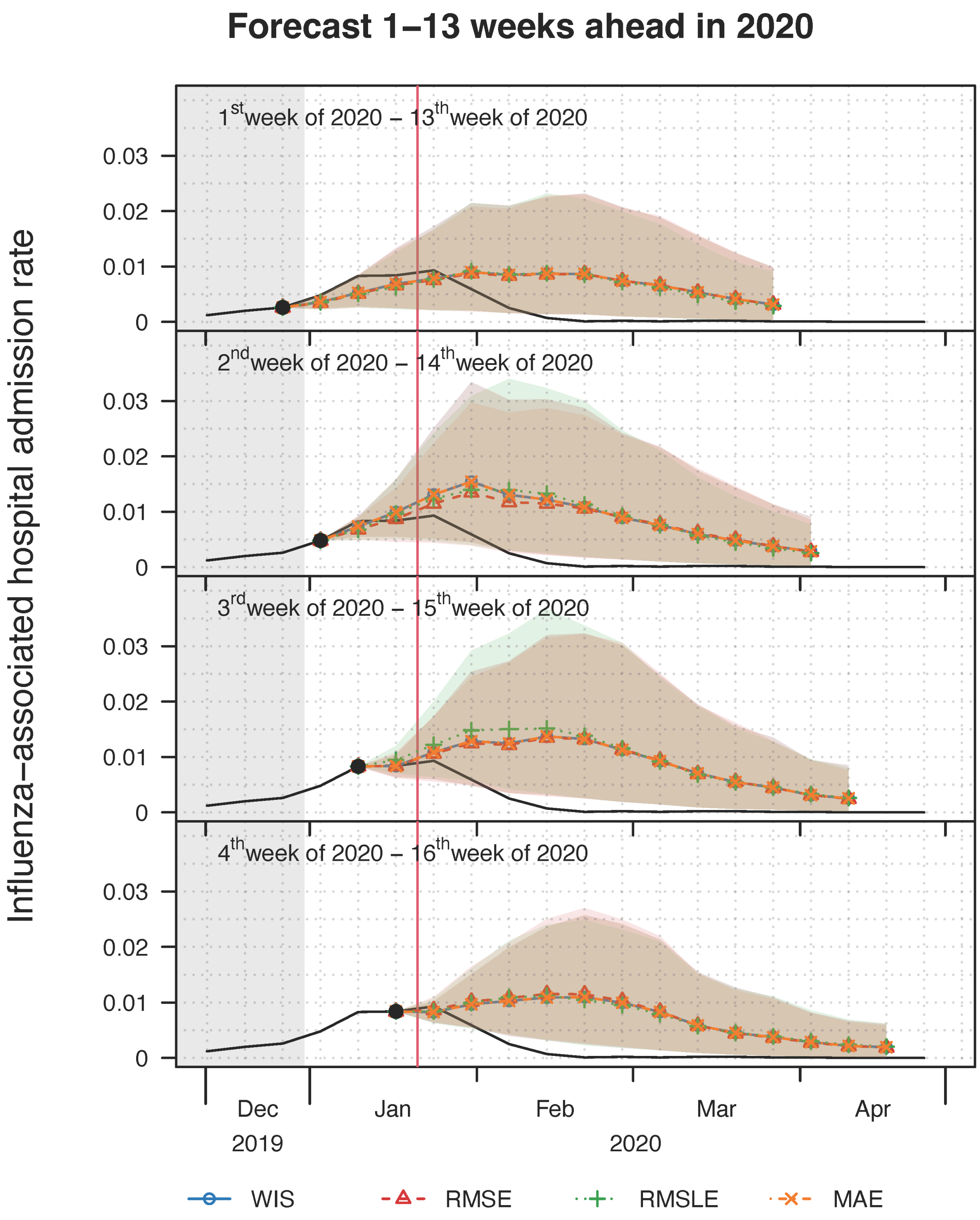

Fig H. Statistical framework-based medium-term (1-13 weeks ahead) forecast on influenza-associated hospital admission rates with different start points of forecast weeks in Hong Kong. We considered by 2nd week of January 2020, there were changes in the population behaviour at individual and community levels and the implication of PHSMs became much effective (1), therefore we started forecast from the following weeks (for main text) and for sensitivity we consider forecasting from 1st to 4th week of January 2020. The black line is the observed influenza-associated hospital admission rate and coloured dashed lines (with respective shapes for different measures of predictive model evaluation) are the admission rate forecast with 95% PI (in respective shades). The black dots indicated the previous week of the start points of forecast weeks and the periods specified at the top-left corner of each panel were the forecast period. The vertical line in red indicates that the first COVID-19 case was identified in Hong Kong on 22nd January 2020.

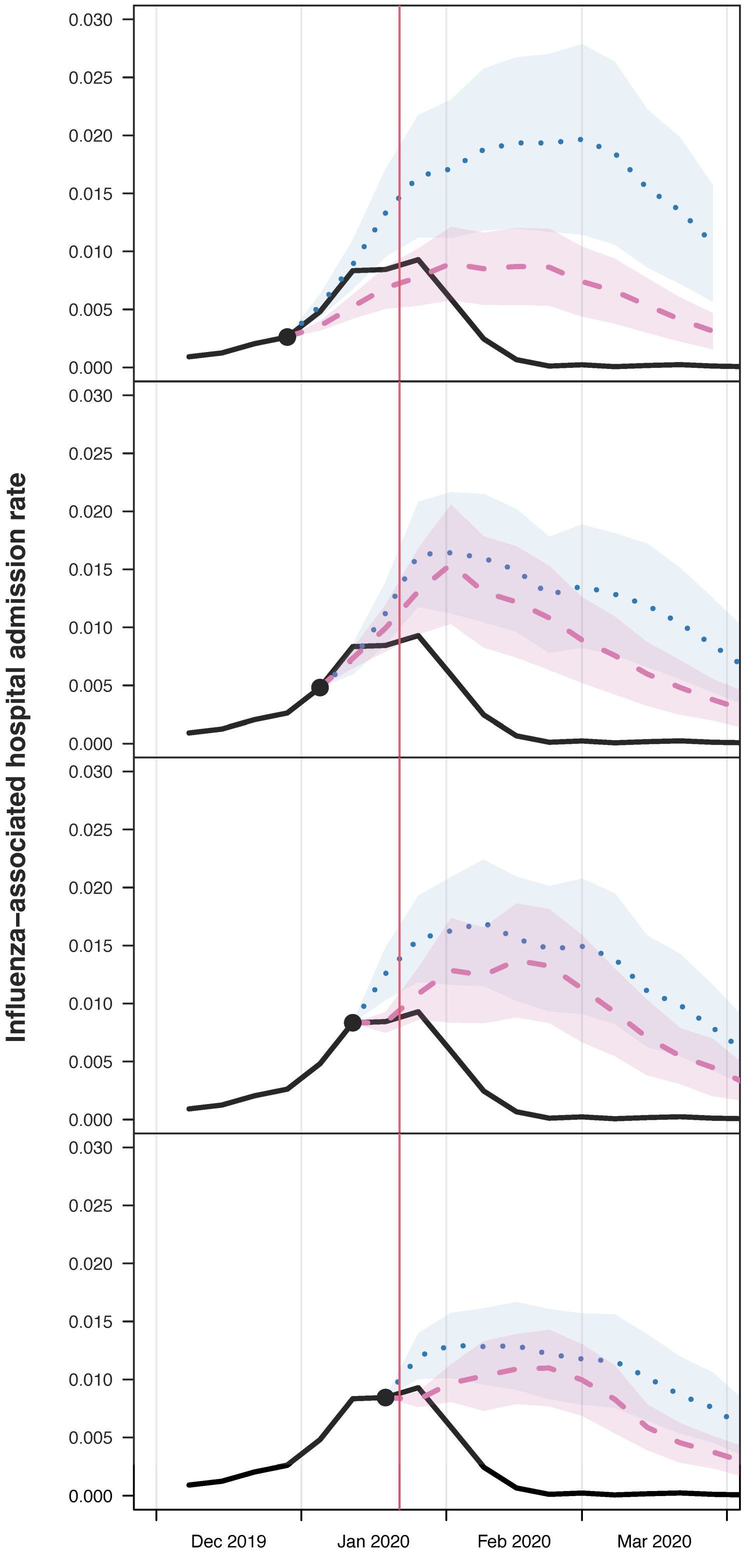

Fig I. Comparison between forecasted hospitalization admission rate by scaling the forecasted influenza activity and by direct forecast, with different start points of forecast weeks in Hong Kong. We considered by 2nd week of January 2020, there were changes in the population behaviour at individual and community levels and the implication of PHSMs became much effective (1), therefore we started forecast from the following weeks (for main text) and for sensitivity we consider forecasting from 1st to 4th week of January 2020. The black line is the observed influenza-associated hospital admission rate. The black dots indicate the previous week of the start points of forecast weeks Blue dotted line is the forecasted hospitalization admission rate by scaling the forecasted influenza activity, while the pink dashed line is the direct hospitalization admission rate forecast. The coloured regions are the 95% confidence interval. The vertical line in red indicates that the first COVID-19 case was identified in Hong Kong on 22nd January 2020.

REFERENCES

1. Cowling BJ, Ali ST, Ng TWY, Tsang TK, Li JCM, Fong MW, et al. Impact assessment of non-pharmaceutical interventions against coronavirus disease 2019 and influenza in Hong Kong: an observational study. Lancet Public Health. 2020;5(5):e279-e88.
