# Supplementary material for: Forecasting of influenza activity and associated hospital admission burden and estimating the impact of COVID-19 pandemic on 2019/20 winter season in Hong Kong
Source: PLoS Comput Biol. 2024 Jul 31;20(7):e1012311. doi: 10.1371/journal.pcbi.1012311 (PMC11318919; doi:10.1371/journal.pcbi.1012311)
Supplement: Attachment — Submitted filename: Point-by-point responses R1.docx [file pcbi.1012311.s002.docx]

**Title: Forecasting of influenza activity and associated hospital admission burden and estimating the impact of COVID-19 pandemic on 2019/20 winter season in Hong Kong (PCOMPBIOL-D-23-02063)**

**Reviewer's Responses to Questions**

**Reviewer #1: Overall**

***Comment 1.1:*** *This is a nice study question and good evidence for the ability of the framework to forecast. It also has implications for the potential effectiveness of social distancing against influenza transmission. It builds on a sizeable literature for forecasting.*

**Response 1.1:** Thank you for your supportive comments.

***Comment 1.2:*** *What were the models used for the different sections? The results-last organisation of the manuscript didn’t really work, because I couldn’t understand what the models were doing. I did jump ahead to the more technical methods section, but that still wasn’t fully clear. I think the regression framework was drawing in historical averages from prior seasons and adjusting those for more recent patterns for the short term forecasts, but allowing the model to regress back to historical averages for medium-term predictions. This is a nice framework and appropriate, but it is difficult for the reader to appreciate this in the current draft. I’d suggest adding in some natural language at the end of the introduction and keeping the current structure, or making the methods more accessible and less technical and switching to methods-first.*

**Response 1.2:**  Thank you for the comments. The models for short/medium-term forecast of influenza activity were listed in Table 1, which were those in bold with the lowest weighted interval score (WIS). Other models in Table 1 were for reference when the selection criteria was changed to root mean square error (RMSE), root mean square log error (RMSLE), and mean absolute error (MAE). Similarly, the models for short/medium-term forecast of influenza-associated hospital admission were listed in S4 Table. As per the manuscript guidelines of *PLOS Computational Biology,* “Results” section should come after “Introduction”, while “Materials and Methods” should be placed after “Discussion”.

Now, we have briefly mentioned the models in the introduction section by revising the text as follows.

“In this study, we used syndromic and virological multi-stream data to construct a statistical regression-based modeling framework for retrospectively forecast winter influenza activity and associated hospital admission burdens in Hong Kong during the COVID-19 pandemic first, and then evaluated the impact of COVID-19 PHSMs on influenza activity in Hong Kong.”

Also, a sentence included before the respective results as,

“We formulated generalized linear regression models considering various potential driving factors as predictors with their possible forms of associations to forecast the weekly influenza activity in Hong Kong, assuming the weekly ILI+ proxy followed a negative binomial distribution (see MATERIALS AND METHODS section for details).”

***Comment 1.3:*** *The paper suggests that reductions in social mixing less than those used in Hong Kong for COVID would be sufficient to control influenza, if that was their purpose. This seems to be an important additional point that could be made more explicitly in the paper.*

**Response 1.3:** Thank you for the comments. Yes, our result already suggested a significant reduction in influenza attack rate, transmissibility, and influenza-associated hospital admission in the winter-spring period of the 2019/20 season associated with the COVID-19 PHSMs. The timing and stringency of these PHSMs, not only modulated the social mixing but also restricted the forward transmission at individual and population levels in general. Our primary aim of this paper was not to investigate the reduction in ILI activity under different levels of reduction in social mixing in particular. Therefore, we didn’t claim these reductions were due to the reduction in social mixing only, rather we reported the overall impact of the PHSMs on influenza.

***Comment 1.4:*** No curated data are available nor is the code available. Code on request is not sufficient.

**Response 1.4:** Thank you for the suggestion. The codes are available at Github: <https://github.com/DanielYCLau/HKFluForcast2020/>. We now revised the text by mentioning the availability of such codes in the section “Data availability”.

***Comment 1.5:*** *Detailed comments. Line 26: make it clear if / whether this was a retrospective study*

**Response 1.5:**  Thank you. We have revised the text in the abstract as follows.

“The objective of this study was to retrospectively forecast influenza activity during the year 2020 and assess the impact of COVID-19 public health social measures (PHSMs) on influenza activity and hospital admissions in Hong Kong”.

***Comment 1.6:*** *89: understanding these factors should help. The authors cite many studies, but often including these factors doesn’t make a massive improvement.*

**Response 1.6:** Thank you. We acknowledged that the inclusion of the potential factors for influenza forecast might not always result in a substantial improvement in accuracy. The impacts of these factors are not even found to be the same for short-term and long-term forecasting. Nonetheless, the purpose of referencing the aforementioned studies was to present a comprehensive overview of the mechanism by which those factors exerted their influence on influenza activity, hence elucidated their impact on influenza forecasting. Those studies provided valuable insights into the complex nature of influenza dynamics, hence also suggested the difficulties associated with influenza forecast.

***Comment 1.7:*** *109: “usually coincide” suggest a proof for grammar*

**Response 1.7:** Thank you for the suggestion. We have revised the text as follows and made a thorough proof check for the manuscript.

“Besides, the summer holidays mostly aligned with the summer peaks of influenza activity and hospitalizations in Hong Kong.”

***Comment 1.8:*** *121: either here or in the introduction, it would be good to discuss measures of forecast accuracy. Different potential customers require different outputs. Here, the forecast is being used as a scientific tool to some degree, but a bit of discussion is warranted as to why we care… perhaps calling out the categorical flood-like levels that are often used by public health professionals. Also worth having a visual comparison of accurate and inaccurate forecasts so that the reader can judge more easily the meaning of the different statistics presented in the table.*

**Response 1.8:** Thank you. Please refer to the response for comments 1.2, it is the requirement of the journal for manuscript structure: introduction, results, and discussion sections followed by materials and method section. We currently revised the text mentioning the modeling framework for the forecast in the introduction and included some text for the respective result section. The model selection criteria were based on weighted interval score (WIS), which has its advantage over the traditional forecast accuracy measure such as RMSE (see the ‘materials and method’ section for details). The respective prediction performance of these models on influenza activity based on time series cross-validations is presented in table 1. Yes, we did visualize the comparison of the selected model based on different criteria in terms of the association of predictors on predictions (Fig S1,S4) and forecast (Fig S3,S5,S7,S8), and the results were found to be comparable.

***Comment 1.9:*** *162:I find the R^2 a difficult measure with which to judge forecast accuracy. Can the authors comment a little on this so that the reader knows how to interpret it. Or change the narrative to focus on one of the measures that are more easily comparable across different forecasting studies. RMSRE at 2 weeks is a pretty good one?*

**Response 1.9:** Thank you for the comments. We used WIS instead of R^2^ for model selection for forecast accuracy because WIS considered both the error in point predictions and prediction intervals which provided a probabilistic perspective on forecast accuracy, while R^2^ only focus on error in point predictions. However, we considered the performance of the whole forecast window for model selection, whereas the performance could be different across each week within the forecast window. Herein the goodness-of-fit (i.e. coefficient of determination, R^2^) was used just to illustrate the forecast accuracy at different weeks ahead given the data at the current time point. Technically, R^2^ is the proportion of variances in observations that the predicted outcomes could explain (1 indicates perfect prediction), hence, the prediction performance at different weeks ahead can be standardized and allow comparison. In contrast, WIS itself was not a standardized measure for such comparison and thus R^2^ was used instead.

***Comment 1.10:*** *180: this is a really nice exposition of a principled counterfactual. The authors should comment a little more on the need for those for historical events when setting policy options for future scenarios.*

**Response 1.10:** Thank you for the comment. Yes, we explored the counterfactual forecasts for the influenza activities and hospital burden during the 2019-20 winter season in Hong Kong to assess the impact of COVID-19 PHSMs on influenza. We already discussed this advancement as an implication of this forecast framework for prospective forecasting in early detection of the upcoming influenza outbreak outcomes. We revised the text as follows to illustrate these implications further.

“The potential implications of our forecasting framework could lead to early detection of the upcoming influenza outbreak in the short term. The models showed satisfactory goodness-of-fit for short-term prediction of 1-4 weeks with the R2 ranging 0.50 - 0.90 (S2 Fig). This allowed our model to provide more accurate forecasts of influenza activity for the coming month and to inform timely disease control measure if an upcoming outbreak is detected in advance. Yet, the low R2 for the prediction beyond 4 weeks limited the confidence of medium-term forecasts, unless different predictors were allowed to be responsible for the prediction of each week ahead at the expense of model interpretation. Therefore, along with the impact assessment of COVID-19 PHSMs via counterfactual retrospective forecasting, the data-driven framework could provide better real-time mitigation policy. The framework could conveniently be extended to prospective forecast the influenza activity and could be efficiently adopted for other directly transmitted diseases in Hong Kong and other locations.”

***Comment 1.11:*** *247: Is this some sense of overshoot? So if the infection becomes well seeded but can’t grown exponentially during school closure it then accelerates more and overshoots more than it would have done had schools been open when it was initially growing? There might be a nice effect to try to tease out here a little more in the discussion if the author can dig out a few references on overshoot?*

**Response 1.11:**  Thank you. A study conducted in eight European countries revealed a reduction in the basic reproduction number during weekends and holidays (Hens N. et al BMC inf Dis (2009)). However, the study also found that intergenerational mixing contacts were more frequent during these periods, while same-age mixing became less frequent. Similar mixing pattern was also observed during the influenza A/H1N1 pandemic (Miller J.C. et al PloS One (2010)), and during COVID-19 pandemic (Andrejko KL et al BMC inf Dis (2022)). This dynamic presented the potential for the emergence of complex epidemiological patterns, where a decrease in the incidence among children was partly offset by an increase in incidence among adults.

We have included the related discussion as following in the revised version as follows.

“This might illustrate the possible increase in influenza activity when the school resumed classes, or due to the increased social interactions during holiday gatherings. Despite the reduction in reproduction number during weekends and school holidays, it was found in European countries that intergenerational mixing contacts were more frequent during these periods, while same-age mixing became less frequent (36). A similar mixing pattern was also observed during the influenza A/H1N1 pandemic (37), or even during the COVID-19 pandemic despite a different context with the significantly stricter PHSMs in place (38). This findings suggested that the decline in the incidence among children was mitigated to some extent by a corresponding rise in the incidence rate among adults.”

- Hens, N., Ayele, G.M., Goeyvaerts, N. et al. Estimating the impact of school closure on social mixing behaviour and the transmission of close contact infections in eight European countries. BMC Infect Dis 9, 187 (2009). <https://doi.org/10.1186/1471-2334-9-187>
- Miller JC, Danon L, O'Hagan JJ, Goldstein E, Lajous M, Lipsitch M (2010) Student Behavior during a School Closure Caused by Pandemic Influenza A/H1N1. PLoS ONE 5(5): e10425. <https://doi.org/10.1371/journal.pone.0010425>
- Andrejko, K.L., Head, J.R., Lewnard, J.A. et al. Longitudinal social contacts among school-aged children during the COVID-19 pandemic: the Bay Area Contacts among Kids (BACK) study. BMC Infect Dis 22, 242 (2022). <https://doi.org/10.1186/s12879-022-07218-4>

***Comment 1.12:*** *277: this implies a maximum R value for flu during that period, which is probably worth reporting*

**Response 1.12:** We have reported the effective reproduction number (Re) based on the observed ILI activity and counterfactual scenario given the forecast result, hence the corresponding reduction in Re in S3 Table. The reduction in Re, which was a measure of the impact of COVID-19 PHSMs on influenza activity, was reported in main text under subheading “Performance on medium-term prediction and forecast of influenza activity”.

***Comment 1.13:*** *310: are there any other studies of forecasting healthcare demand in HK?*

**Response 1.13:** Thank you for the suggestion, but unfortunately no such studies reported on healthcare demand for Hong Kong to discuss here.

***Comment 1.14:*** *362: This section needs some natural language added to make it more accessible. Perhaps at the end of the intro or woven in here. Broadly, what signals can the framework pickup? How are they combined and is there a history of this type of framework being good for prediction?*

**Response 1.14:** Thank you for the comments. The framework first ensured to detect the signals of the potential drivers by exploring various plausible forms of these driving factors with the forecasting outcomes, and constructed the predictive models by training a series of models on historical data of these drivers. We have provided the details in ‘materials and method’ section under subheading “Statistical Modelling framework for forecast”. We recognized that GLM as a conventional statistical approach might not yield exceptionally high levels of prediction or forecast accuracy (Dugas AF et al PLoS ONE (2013)). In contrast, machine learning (ML) models have gained popularity due to their ability to effectively model complex systems with high accuracy, meanwhile GLM often serves as one of the candidate models for comparative analysis in infectious disease forecasting research (Xu Q et al PLoS ONE (2017), Darwish A et al BMC Res Notes (2020)). Nevertheless, ML models are often characterized by their limited interpretability, whereas GLM offers the advantage of interpretability and statistical inference. Further, the final results and intermediate results under GLM are directly interpretable for epidemiological events or outcomes. Consequently, in our study, we adopted GLM to strike a balance between interpretability and accurate results.

In revised version we further included the predictive models by ARIMA for comparative modelling performance (please refer to the response for the comment 2.3).

- Dugas AF, Jalalpour M, Gel Y, Levin S, Torcaso F, Igusa T, et al. (2013) Influenza Forecasting with Google Flu Trends. PLoS ONE 8(2): e56176. <https://doi.org/10.1371/journal.pone.0056176>
- Xu Q, Gel YR, Ramirez Ramirez LL, Nezafati K, Zhang Q, Tsui K-L (2017) Forecasting influenza in Hong Kong with Google search queries and statistical model fusion. PLoS ONE 12(5): e0176690. <https://doi.org/10.1371/journal.pone.0176690>
- Darwish, A., Rahhal, Y. & Jafar, A. A comparative study on predicting influenza outbreaks using different feature spaces: application of influenza-like illness data from Early Warning Alert and Response System in Syria. BMC Res Notes 13, 33 (2020). <https://doi.org/10.1186/s13104-020-4889-5>

**Reviewer #2: General:**

***Comment 2.1:*** *This is a well-written manuscript, which investigates the ability to forecast influenza epidemics in a subtropical region via a traditional generalized linear modeling approach. However, I have a couple of concerns, which should be addressed before publication.*

**Response 2.1:** Thank you for the supporting comments.

***Comment 2.2:*** *Major: 1. The authors should better highlight the novelty of their work. It does neither become clear what is new here from an application point of view nor from a methodological perspective.*

**Response 2.2:** In terms of advancement of the study, we already have discussed in the text (discussion section) by illustrating the implication of forecasting exercise for assessing the impact of COVID-19 PHSMs on infulenza activities in Hong Kong. This is perhaps the first study to consider retrospective counterfactual forecast to assess the impact of the PHSMs on respiratory disease like influenza during pandemic. The framework can be generalised the for other respiratory disease and other locations for even prospective forecasting and now-casting of the outcomes.

We also revised the text in introduction (see the response for the comment 1.2) to mention the modelling framework used in the study. In theory, GLM offered a straightforward and adaptable forecasting framework with interpretability and statistical inference in our study. In contrast to the Gaussian error assumption made by ARIMA model, GLM allowed for modelling of non-Gaussian response variables by specifying an appropriate distribution and link function (e.g. negative binomial distribution with a log link), enhancing flexibility in capturing the relationship between predictors and the response variable. Moreover, coefficients corresponding to specific predictors in GLM facilitated the interpretation of associations between predictors and the response variable. Additionally, the statistical properties of GLM enabled the direct derivation of forecast intervals. Conversely, machine learning (ML) models achieved high prediction/forecast accuracy at the expense of interpretability of predictors’ associations. Iterative training on quantiles was also required for ML model to derive the forecast interval. Further, these predictive models are constructed by training on historical multi-stream data including syndromic, virological, social, control measures during the study.

***Comment 2.3:*** *2. The authors propose a GLM based modeling approach, assuming ILI+ to follow a negative binomial distribution. No justification is given for this choice. Furthermore, there is no comparative modeling approach such as ARIMA tested.*

**Response 2.3:** Thank you for the comment and suggestion. The data for model fitting was count data as we further scaled up the influenza activity proxy to approximate the incidence number of influenza infections in a million consultation. In theory, such case count usually represented by Poisson (in general negative binomial) process. Therefore, Poisson and negative binomial distribution are the common choices to model count data for GLM. While Poisson distribution assumes variance is the same as mean, negative binomial distribution has a parameter to control for overdispersion in case the variance is larger than the mean in the data, and it can reduce back to Poisson distribution if the data is not overdispersed. Thus, negative binomial distribution is employed in our study given its flexibility, and the value of overdispersion is obtained by estimation. We included this methodological details in the main text under subheading “Statistical Modelling framework for forecast” as follows.

“Besides, an overdispersion parameter ($\phi_{k}$) was introduced to control the variance for the forecast at $k$-th week, and the forecast would reduce to be Poisson-distributed if there was no overdispersion.”

In revised version, we have included the ARIMA model based forecast and compared the forecasting performance with GLM. We added a section in ‘material and method’ section under ‘Sensitivity analysis with Autoregressive integrated moving average (ARIMA) models based forecasting’ . The related results are also presented in sub-section ‘Sensitivity analysis with alternative models (ARIMA models) for comparing forecast performance’ and in additional tables S7-S8 and discussed (lines 325-328). We noticed, in general, GLM performed better than ARIMA model.

***Comment 2.4:*** *3. Time series cross-validation is non-trivial. Based on the description on pages 19/20 it remains unclear, how exactly training and test sets were constructed. Was a sliding training window used? If yes, which size did it have and which stride? Was an expanding window applied? Was their a gap between training and test set?*

**Response 2.4:** Thank you for the valuable suggestion. The previous result was based on traditional 10-fold cross-validation (CV) method, and now the result was updated based on time series CV. In brief, we split the data into training and testing set under a 70/30 ratio (i.e. training window of 7 years for influenza activity), and the time series CV was conducted in a rolling approach with the stride of 1 week. There was no gap between training and test set. In the updated result, the ozone concentration school holiday were also selected as the predictors with high forecast power, and absolute humidity may be switch to mean temperature to provide higher forecast power as they are highly correlated. The estimates were similar in terms of short-/medium-term forecast in 2020 and hence the reduction associated with COVID-19 PHSMs.

We have updated the result based on time series CV, and revised the text in sub-section “Short-term prediction and Forecast of influenza activity” of the ‘material and method’ section as below.

“To assess the prediction performance of the models for forecasting of influenza activity, we conducted a time series cross-validation during the period 2010-2019 for model selection. Under a 70/30 training-to-testing ratio, we used data from January 2017 to December 2019 as testing period, hence we fixed the training period for 7 years (i.e from January 2010 to December 2016 as the first training window) and evaluated the data in the coming $k$ weeks (i.e. 1st- $k$th week in 2017 as the first testing window) using a rolling window approach.”

***Comment 2.5:*** 4*. The authors evaluated the statistical influence of public health measures on model predictions. However, this does not tell us anything about the causality of those measures. This point should be emphasized more.*

**Response 2.5:** Thank you. The framework is based on the data-driven model prediction and forecasting of influenza activity. Such ecological framework usually did evaluate the causal relationship between the predictors and outcomes directly. Further control studies and experimental design were required to provide stronger evidence of the causality between them. We discussed this in the revised text as an additional limitation as follows.

“Finally, our result did not imply causal relationship between the predictors and influenza activity. Our statistical model could investigate the forecast performance of potential predictors on influenza activity by their correlation, whereas further studies and experimental design were required to provide stronger evidence of the causality between them.”
